# Deficiency in *Aim2* affects viability and calcification of vascular smooth muscle cells from murine aortas and angiotensin-II induced aortic aneurysms

**DOI:** 10.1186/s10020-020-00212-z

**Published:** 2020-09-15

**Authors:** Markus Wortmann, Muhammad Arshad, Maani Hakimi, Dittmar Böckler, Susanne Dihlmann

**Affiliations:** 1 Department of Vascular and Endovascular Surgery, University Hospital Heidelberg, University of Heidelberg, Im Neuenheimer Feld 110, 69120 Heidelberg, Germany; 2 Present Address: Department of Vascular Surgery, Luzerner Kantonsspital, Spitalstrasse, 6000 Luzern 16, Switzerland

**Keywords:** VSMC, Phenotype transition, Inflammasome, Senescence, Proliferation, Aortic aneurysm

## Abstract

**Background:**

Phenotypic transformation of vascular smooth muscle cells is a key element in vascular remodeling and aortic aneurysm growth. Previously, deletion of several inflammasome components decreased formation of aortic aneurysm (AA) in the Angiotensin II (AngII) -induced mouse model. We hypothesized that the inflammasome sensor Absent in melanoma 2 (Aim2) might affect the phenotype of vascular smooth muscle cells (VSMC), thereby reducing AA formation.

**Methods:**

*Aim2−/−* mice and wild-type (WT) C57Bl/6 J mice were used as an animal model. VSMC were isolated from 6 months old mice and grown in vitro. Young (passage 3–5) and senescent (passage 7–12) cells were analyzed in vitro for calcification in mineralization medium by Alizarin Red S staining. Expression of calcification and inflammatory markers were studied by real-time RT-PCR and Western blotting, release of cytokines was determined by ELISA. To induce AA, osmotic mini-pumps loaded with AngII (1500 ng/kg bodyweight/min) were implanted for 28 days in male mice at 6 months of age.

**Results:**

Compared with VSMC from WT mice, VSMC isolated from *Aim2−/−* mice were larger, less viable, and underwent stronger calcification in mineralization medium, along with induction of *Bmp4* and repression of *Tnfsf11/Rankl* gene expression. In addition, *Aim2* deficiency was associated with reduced inflammasome gene expression and release of Interleukin-6. Using the mouse model of AngII induced AA, *Aim2* deficiency reduced AA incidence to 48.4% (15/31) in *Aim2−/−* mice versus 76.5% (13/17) in WT mice. In contrast to *Aim2−/−* mice, AA from WT mice expressed significantly increased levels of alpha-smooth muscle actin/*Acta2*, indicating tissue remodeling. Reduced cell proliferation in *Aim2−/−* mice was indicated by significantly increased p16ink4a/*Cdkn2a* expression in untreated and AngII-infused aortas, and by significantly lower amounts of proliferating (Ki67 positive) VSMC in AngII-infused *Aim2−/−* mice.

**Conclusions:**

Our results suggest a role for Aim2 in regulating VSMC proliferation and transition to an osteoblast-like or osteoclast-like phenotype, thereby modulating the response of VSMC in aortic remodeling and AA formation.

## Background

Phenotype transformation of vascular smooth muscle cells (VSMC) is considered a driving force of many vascular diseases, including aortic aneurysms (Bennett et al. 2016; Hortells et al. 2018; Riches et al. 2018). Human abdominal aortic aneurysm (AAA) is an age-related disease, defined by dilation of the aorta by more than half of the original diameter. During aneurysm growth, the aortic wall is extensively remodeled and weakened, thereby increasing the risk of rupture, which results in massive, and often fatal internal bleeding (Reimerink et al. 2013; Sampson et al. 2014). Current clinical interventions are associated with significant morbidity and mortality, and sometimes surgery is not possible at all. Therefore, there is an unmet clinical need for development of a conservative medical treatment to limit or prevent progression of small AAAs by specifically targeting the vascular remodeling processes.

The sequential pathogenesis and detailed biological mechanisms underlying AAA formation are incompletely understood. Remodeling of the aortic wall is accompanied by invasion of inflammatory and immune cells, extensive alterations in the extracellular matrix composition and considerable changes of VSMC phenotype (Ailawadi et al. 2009; Golledge 2019; Petsophonsakul et al. 2019). In the healthy aorta, most VSMC display a quiescent contractile phenotype, defined by expression of contractile proteins such as alpha-smooth muscle actin (αSMA) and smooth muscle myosin II, which allows them to maintain vascular tone. In response to physical or biochemical stress factors that accumulate with ageing, VSMC have the ability to switch to synthetic phenotypes. Synthetic VSMC are characterized by a decreased expression of contractile proteins and increased expression of extracellular matrix degrading enzymes, inflammatory cytokines and/or calcification-promoting genes (Owens et al. 2004). In addition, multipotent vascular stem cells, residing in the vessel wall appear to differentiate into VSMC in response to injury and adapt myofibroblast-like, macrophage-like, or osteoblast-like phenotypes (Tang et al. 2012). Today, VSMC phenotypic switching and calcification are considered key in AAA formation (Petsophonsakul et al. 2019; Riches et al. 2018) and the phenotypic modulation has been shown to be an early event in the aorta before aneurysm growth (Ailawadi et al. 2009).

Using animal and in vitro cell culture models, a number of cellular signaling pathways have been identified, which regulate VSMC transition in aortic aneurysm formation (reviewed in (Petsophonsakul et al. 2019) and (Golledge 2019)):

(1) BMP- and Wnt/β-Catenin-Signaling have been implicated in regulating osteochondrogenic differentiation of VSMC under calcifying conditions and to promote angiotensin II (AngII) induced aortic aneurysm (AA) (Freise et al. 2016; Krishna et al. 2017; Towler 2017). (2) Components of the inflammasomes, which mediate Caspase-1 dependent activation of interleukin 1β (IL-1β) and subsequent inflammatory cascade, have been shown to contribute to VSMC transformation and aortic aneurysms (Johnston et al. 2013, 2014). NLR family pyrin domain containing 3 (NLRP3) is required for VSMC phenotypic transformation and calcification (Ren et al. 2017; Sun et al. 2017; Wen et al. 2013). In addition, genomic inactivation of the inflammasome components *Nlrp3*, *Casp1* (Caspase-1) and *Apc* (Apoptosis-associated speck-like protein containing a caspase recruitment domain) reduced the development of AngII-induced aortic aneurysm by inhibiting IL-1β release from bone marrow derived macrophages (Usui et al. 2015).

Absent in melanoma 2 (AIM2) is another well-described inflammasome component, that has recently been implicated to play a role in human AAA formation (Dihlmann et al. 2014; Hakimi et al. 2014; Wortmann et al. 2019b; Wu et al. 2016) and other inflammatory diseases (Sharma et al. 2019). In response to cytoplasmic double stranded (ds) DNA, AIM2 activates an inflammasome in macrophages (Fernandes-Alnemri et al. 2009; Hornung et al. 2009). Its role in other vascular cell types is less well defined, although we could recently show that human VSMC derived from AAA-patients respond to necrotic cell debris with an induction of AIM2 (Wortmann et al. 2019a). Given this observation and the above-mentioned association of inflammasome activity with AAA and calcification, we aimed to further investigate the role of AIM2 in murine VSMC and AA formation. We hypothesized that the VSMC phenotype, particularly calcification in response to ageing and/or AngII, might be altered by AIM2. To test this hypothesis, we used VSMC cultures isolated from *Aim2−/−* (C57Bl/6 J background) and WT (C57Bl/6 J) mice. Furthermore, we aimed to test whether an *Aim2* knockout protects from AA development in the well-established murine AngII-induced model, whereby AA formation is triggered by continuous subcutaneous application of AngII to mice. To further identify the molecular basis for these effects, VSCM cultures, isolated from AngII-induced mice were also analyzed for phenotypic changes. The findings obtained in this study provide insight into the pathophysiology of AA, suggesting that AIM2 may be added to the therapeutic targets for preventing AA progression.

## Methods

### Animal models

All experiments in this study were performed in accordance with federal law for animal protection and approved by the regional council of Baden-Wuerttemberg (Regierungspraesidium Karlsruhe). *Aim2−/−* mice (B6.129P2-*Aim2*^*Gt(CSG445)Byg*^/J) and wild-type (WT) C57Bl/6 J mice (JAX™ C57BL/6 J), used as a control, were purchased from Charles River (Sulzfeld, Germany). All mice used in this study had a C57Bl/6 J genetic background. Male mice at 6 months of age were chosen for our experiments to model the epidemiological background in humans, where AA is associated with increased age and male sex (Forsdahl et al. 2009; Hannawa et al. 2009). Mice were kept in a pathogen-free animal facility (IBF Heidelberg) with standard rodent food and tap water ad libitum. To induce AA, osmotic mini-pumps (Model 2004, Alzet Scientific products, Cupertino, CA) loaded with AngII (1500 ng/kg bodyweight/min) were implanted in the dorsal subcutaneous space for 28 days in male mice at 6 months of age. During surgery, for performing regular ultrasound examinations, and at the end of the 4-week experiment, mice were anesthetized with isoflurane.

### Assessment of AA formation

Maximum aortic diameter was determined on a weekly basis by ultrasound. In absence of an aneurysm, maximum aortic diameter was measured at the entry into the abdominal cavity at the level of the diaphragm. Dilation of the aortic diameter to > 1.5 mm was defined as an aneurysm. In this case the maximum diameter of the aortic aneurysm was measured. In addition, aortic aneurysms were histologically confirmed post-mortem using serial sections of formalin-fixed paraffin-embedded aortas.

### VSMC cell culture

VSMC were isolated from aortas of 6 months old male mice, infused for 28 days with AngII, or from age-matched control animals. Aortas were cut up into small pieces and digested with 400 U/ml collagenase type II (Worthington, Biochemical Corporation, Lakewood, USA) for 1–2 h at 37 °C with shaking. Isolated cells were washed with PBS and expanded in 6-well plates containing Smooth Muscle Cell Growth Medium 2 (PromoCell, Heidelberg, Germany), supplemented with antibiotics (100 U/ml penicillin/streptomycin and 5 μg/ml amphotericin B) in a humidified atmosphere of 5% CO_2_. Cells were pooled from 2 to 3 animals. The media was replaced every 3 days. Subcultures were obtained by using 0.05% Trypsin/EDTA (Life Technologies, Gibco, Paisley, UK) and cells were split 1:2 when reaching 70–90% confluence. Identification of vascular smooth muscle cells was performed by visual inspection of morphology and growth in hills and valleys, and the cells were analyzed at different passages for expression of alpha smooth muscle actin. Cells were considered young for the first 3–5 passages and used for experiments with proliferating cells. To obtain replicative senescent cells, VSMC were split regularly in Smooth Muscle Cell Growth Medium 2 until they stopped growing for more than 2 weeks.

### Analysis of the number of passages

A subset of each VSMC culture was grown in six-well plates, and cells were split 1:2 when reaching 90–100% confluence. Senescence was defined as complete growth cessation for more than 2 weeks.

### Cell proliferation / viability assay

VSMC were seeded at a density of 1000 cells per well in a 96well plate in triplicates. Serum starvation was performed over night by reducing the medium supplement to 10% of the amount that was required for regular growth (Smooth Muscle Cell Growth Medium 2; PromoCell, Heidelberg, Germany). After 24 h, the starvation medium was replaced by full medium (100 μl per well) and cells were grown for an additional 0 h, 24 h, 48 h, 72 h and 96 h. At the different time points, 10 μl per well of WST-1 reagent (Roche Diagnostics GmbH, Mannheim Germany) was added to the cells for 30 min. in a humidified atmosphere at 37 °C and 5% CO_2_. Absorbance of the samples was measured against a background control using a microplate reader at a wavelength of 450 nm and a reference wavelength of 620 nm.

### Induction and quantification of VSMC in vitro calcification

Cells were shifted to Osteoblast Mineralization Medium (PromoCell, Heidelberg, Germany) for 14 or 21 days, with medium change every 3 days to induce calcification. VSMC were washed three times with PBS and calcium deposition was visualized and quantified by Alizarin Red S (ARS staining quantification assay, Sciencell Research Laboratories, San Diego, CA, USA) as recommended by the manufacturer. Briefly, cells were fixed in 4% formaldehyde for 15 min. at room temperature and washed three times with distilled H_2_O. One ml of 40 mM ARS was added per well and incubated at room temperature for 30 min. With shaking. After washing of the cells five times with distilled H_2_O, images were taken using a microscope. Plates were stored at − 20 °C prior to dye extraction. For quantification, 800 μl of 10% acetic acid was added to each well of the 6-well plate for 30 min. Cells were collected using a cell scraper and transferred to a microcentrifuge tube. After vortexing for 30 s, samples were heated at 85 °C for 10 min. Samples were incubated on ice for 5 min and centrifuged at 20000 g for 15 min. Supernatants were transferred to a new tube and 200 μl of 10% ammonium hydroxide was added. Absorbance of the samples was read at 405 nm with a plate reader and plotted against an ARS standard curve to determine the concentration.

### RNA extraction and quantitative real-time polymerase chain reaction (qRT-PCR)

Extraction of total RNA, using Qiagen RNase Mini Kit from cells, and real-time PCR (with an ABI StepOnePlus reader) was performed as described previously (Wu et al. 2015). Primer sequences are listed in supplementary table 1. Relative expression was determined from cycle thresholds (C_T_) by using individual standard amplification curves of each transcript relative to the corresponding mean expression of three reference transcripts (mouse genes: *Gapdh, B2m, Actb*).

### Immunoblot analysis

Cells were harvested in cell lysis buffer and homogenized by ultrasound as described previously (Wu et al. 2016). Twenty μg of each lysate were separated by SDS-PAGE and blotted onto nitrocellulose. Detection of proteins was performed over night with primary antibodies diluted in appropriate buffers as recommended by the manufacturers. Anti-CDKN2A/p16ink4a (EPR20418, No. ab211542; 1:2000) was purchased from Abcam. Mouse-specific antibodies against α-smooth muscle actin (D4K9N, No. 19245; 1:1000), Phospho-Histone H2A.X (Ser139, No. 9718; 1:1000), Phospho-NF-kB p65 (Ser536, No. 3033; 1:1000) and  GAPDH (14C110; No. 2118; 1:1000) were all purchased from Cell Signaling Technology, Danvas, MA, USA. Anti-rabbit IgG HRP-linked secondary antibody (No. 7076, Cell Signaling Technologies; 1:2000) and Western Bright ECL (Advansta, Menlo Park, CA, USA) were used for signal detection by chemoluminescence.

### Quantification of cytokine release into cell supernatant

Supernatants derived from cells were analyzed for concentrations of active cytokines and chemokines according to the recommendations of the manufacturers. For analysis of cleaved IL-1β (p17) the Duo-Set ELISA Development system for murine IL-1β (R&D Systems Europe, Abington, UK) was used. ELISA kits for analysis of murine IL-6 and sRANKL in the supernatants were purchased from Peprotech, London, UK.

### Histology and immunohistochemistry

Mouse aortas were fixed in 4% formalin, embedded in paraffin and 2–5 μm serial sections were cut using a microtome. Sections were stained with haematoxilin (HE), Sirius Red, or Elastica van Gieson (EVG) according to standard procedures. Detailed protocols are available upon request.

For immunohistochemical analysis, sections were dried overnight, deparaffinised and incubated overnight with specific antibodies detecting alpha-smooth-muscle actin (D4K9N, No. 19245, Cell Signaling Technology), p16ink4a (EPR20418, Abcam ab211542), Ki67 (No. 12202, Cell Signaling Technology) or CD45 (MAB114, Biotechne, R&D Systems). R.T.U. biotinylated anti-rabbit IgG (BP-9100, Vector Laboratories, Burlingame, CA, USA) and R.T.U Vectastain Elite ABC reagent, Peroxidase (R.T.U. Vectastain Kit; PK-7100, Vector Laboratories) were used for detection, as described by the manufacturer. Development was performed with ImmPAct AEC reagent (SK-4205, Vector Laboratories). Sections were counterstained with Mayer’s hemalum solution (1:10). Slides were scanned for digital histology with Aperio ImageScope v12.2.2.5015.

### Statistics

**Fig. 1:**
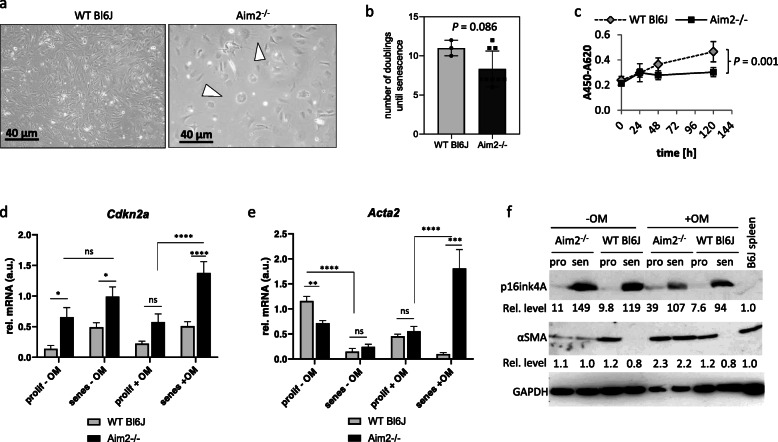
*Aim2* knockout affects the morphology, replicative potential and senescence of murine VSMC. VSMC were isolated from WT and *Aim2*^*−/−*^ mice and pooled. Each pool contained VSMC of 3 animals. **a** VSMC derived from *Aim2−/−* mice are larger, rounded and contain big vacuoles. The picture shows representative cultures of passage 3 from *n* = 6 (*Aim2−/−*) and *n* = 7 (WT) VSMC pools. **b** VSMC derived from *Aim2−/−* mice (*n* = 9 pools) undergo faster replicative senescence than WT VSMC (*n* = 3 pools). **c** Viability of VSMC was determined by WST1-proliferation assay after proliferation of the cells for 24, 48, 72 and 96 h in normal growth medium. **d**-**e** Analysis of mRNA expression of the senescence marker *Cdkn2a/p16* (**d**) and *Acta2* (**e**) was determined by real-time RT-PCR from VSMC grown in normal growth medium or in mineralization medium (OM). Data show the mean and SEM derived from 3 pools of proliferating (passage 3–4) and senescent (passage 7–12) VSMC. Senescence was defined as no proliferation for > 2 weeks. Data were tested by one-way ANOVA. ns: not significant, *: *P* < 0.05; ** *P* < 0.01; ****: *P* < 0.0001. **f** Western blot showing expression of p16ink4A and α-SMA in VSMC pools (derived from *n* = 2–3 animals per pool) of *Aim2−/−* versus WT mice. Numbers represent the relative protein level normalized to GAPDH. Expression level in the spleen of WT mice was set as 1.0

**Fig. 2:**
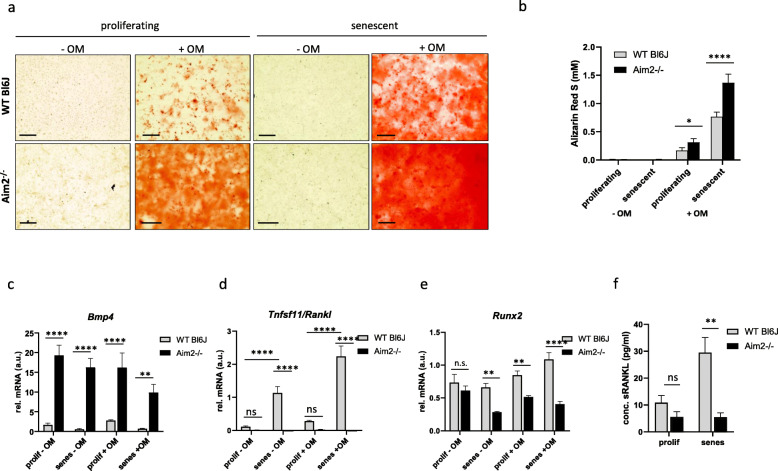
*Aim2* knockout increases calcification of VSMC grown in mineralization medium. **a** Representative images of Alizarin Red S (ARS) stained VSMC. Proliferating and senescent cells were grown in mineralization medium (+ OM) or regular SMC growth medium (− OM). Cells were stained with ARS for visualization in triplicates at day 21. Bar: 40 μm. **b** Quantitative measurement of ARS staining. ARS was eluted from the cells and analyzed photometrically at 405 nm. Bars show the mean and SD from 3 measurements of VSMC pools. **c**-**e** Analysis of mRNA expression of *Bmp4* (**c**), *Tnfsf11/Rankl* (**d**), and *Runx2* (**e**), by real-time RT-PCR. Total RNA was extracted from VSMC grown in vitro with normal growth medium (−OM; *n* = 3 pools) or osteoblast mineralization medium (+ OM; *n* = 3 pools) at day 14. A.u. arbitrary unit; relative expression of target gene was normalized against expression of housekeeping genes (*Actb, Gapdh* and *B2m*). Bars represent the mean and SEM of three measurements from three pools each. **a**-**e** Data were tested by one-way ANOVA. ns: not significant, *: *P* < 0.05, **: *P* < 0.01, ****: *P* < 0.0001. **f** Concentration of murine sRANKL secreted into the supernatant, as determined by ELISA. Data show the mean and SEM of *n* = 3 independent experiments with different VSMC pools for each genotype

GraphPad Prism 8 software (GraphPad, San Diego, CA, USA) was used to perform statistical analyses. Categorical variables (i.e. AA incidence in different mouse genotypes) were analyzed as 2 by 2 contingency tables by Fisher’s exact test. D’Agostino & Pearson test was used to test the data sets (mRNA expression in Figs. 1 and 2) for normal distribution. Statistical significance was tested using one-way analysis of variance (ANOVA) or Student’s t-test as indicated in the figures. The numbers of individuals, cell cultures, independent experiments and other variables used for each test, are indicated in the figure legends. Data are expressed as mean ± SEM or ± SD as indicated in the figure legends. *P*-values of < 0.05 were considered statistically significant.

## Results

### An Aim2 knockout affects the morphology, replicative potential and senescence of murine VSMC

We first investigated effects of *Aim2* deficiency on native VSMC isolated from 6 month (28.4–31.4 weeks) old animals. Examination of early cultures (passage 1–3) by light microscopy demonstrated that VSMC derived from *Aim2−/−* mice reproducibly formed large rhomboidal cells with star-shaped filaments (Fig. 1a), as described for the synthetic phenotype (reviewed in (Petsophonsakul et al. 2019)). In contrast, VSMC derived from WT mice were spindle-shaped and grew densely packed in typical hills and valleys as described for the contractile phenotype (Petsophonsakul et al. 2019). Furthermore, VSMC derived from *Aim2−/−* mice grew slowly with an average doubling time of 3–4 days and reached senescence after 8.3 +/− 2.3 passages (1:2 splitting), whereas VSMC derived from WT mice stopped growing after 11.3 +/− 1.1 passages (Fig. 1b). Accordingly, the viability of VSMC from *Aim2−/−* mice was significantly reduced (Fig. 1c) and expression of the senescence marker *Cdkn2a* (p16ink4a) was significantly higher in early passage (proliferating) and senescent *Aim2−/−* VSMC than in corresponding WT VSMC (Fig. 1d (−OM)). In line with a synthetic phenotype, expression of *Acta2*/αSMA was significantly lower in proliferating *Aim2−/−* VSMC compared with WT VSMC and was further reduced in both genotypes, when the cells reached replicative senescence (Fig. 1e, (−OM)).

### Aim2 knockout promotes osteoblastogenic differentiation of murine VSMC in vitro

Because VSMC phenotypic switching and calcification were shown to be a key event in aneurysm formation (Petsophonsakul et al. 2019), we next asked whether AIM2 is involved in VSMC calcification. To address this query, proliferating and senescent VSMC were shifted to mineralization medium for 2 weeks in vitro. A commercially available osteoblast mineralization medium was chosen for this experiment, because it enables mineralization of existing osteoblast-like precursors. Calcification of VSMC in this culture medium was tested in preliminary experiments with murine VSMC (supplementary figure S1). Staining with Alizarin Red S demonstrated a significantly increased calcification level in proliferating VSMC from *Aim2−/−* mice after shifting to mineralization medium (Fig. 2a, b).

Again, gene expression of the senescence marker *Cdkn2A/p16* was significantly higher in *Aim2−/−* VSMC, compared with WT VSMC, grown for 2 weeks in mineralization medium (Fig. 1d, (+OM)). However, there was no further increase compared with VSMC grown in normal growth medium. Consistent with VSMC phenotype transition, gene expression of *Acta2* was downregulated in WT VSMC grown in mineralization medium, when the cells reached senescence (Fig. 1e, f). Interestingly, both *Acta2* mRNA and corresponding αSMA protein levels were strongly upregulated in senescent *Aim2−/−* VSMC, grown in mineralization medium (Fig. 1e and f, (+OM)).

To further understand the mechanism underlying the increased calcification of *Aim2−/−* VSMC, we investigated expression of a set of genes involved in osteochondrogenic differentiation. A markedly increased expression of *Bmp4* was observed in *Aim2−/−* VSMC (Fig. 2c), whereas expression of the osteoclast differentiation factor *Tnfsf11/Rankl* was completely repressed (Fig. 2d). In line with this, expression of *Runx2*, a transcription factor known to regulate the Rankl promoter was also significantly lower in *Aim2−/−* VSMC, particularly, when the cells reached replicative senescence (Fig. 2e). The expression differences were observed before and after shifting the cells to mineralization medium, indicating that this was an inherent feature of the different genotypes and was not induced by external factors. *Tnfsf11/Rankl* mRNA levels were further increased in WT VSMC reaching senescence but remained absent in *Aim2−/−* VSMC (Fig. 2d). Accordingly, secretion of sRANKL was significantly reduced in *Aim2−/−* VSMC compared with WT VSMC (Fig. 2f). Expression of the osteoblast marker *Bmp2* was particularly reduced in *Aim2−/−* VSMC grown in normal growth medium, whereas this difference disappeared when the cells were shifted to mineralization medium. (Supplementary figure S2a). The mRNA expression of other genes involved in osteogenic differentiation was not associated with the genotype. *Col1A1, Col2A1*, *Mmp9* and *Sox9* expression displayed great variations between individual VSMC cell pools, resulting in marginal or not significant differences between WT and Aim2−/− VSMC (Supplementary figure S2b-e).

### Aim2 deficiency is associated with a reduced inflammatory response of VSMC

**Fig. 3:**
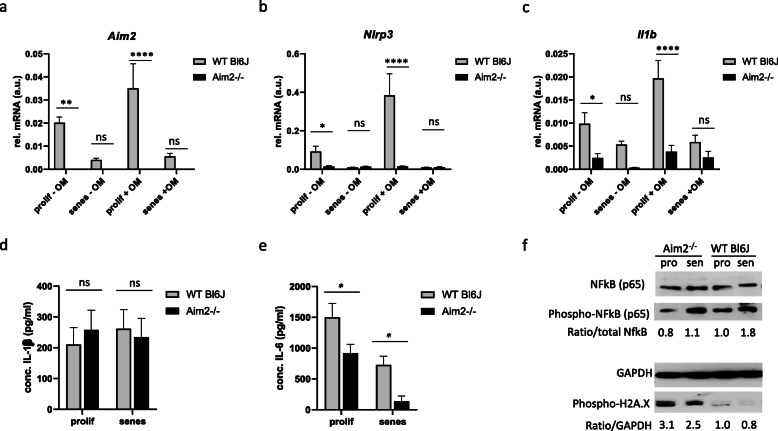
Deletion of *Aim2* gene attenuates the inflammatory response in aortic VSMC of mice. **a**-**c** Analysis of mRNA expression of *Aim2* (**a**), *Nlrp3* (**b**), *Il1b* (**c**), by real-time RT-PCR (*n* = 3 measurements of 3 experiments for each). Total RNA was extracted from VSMC grown in vitro with normal growth medium (−OM) or osteoblast mineralization medium (+ OM) at day 14. A.u. arbitrary unit; relative expression of target gene was normalized against expression of housekeeping genes (*Actb, Gapdh* and *B2m*). **d**, **e** Concentrations of secreted IL-1β (**d**) and IL-6 (**e**) in the supernatant of WT and *Aim2−/−* VSMC was determined by ELISA. Data show the mean and SEM of *n* = 3 independent VSMC pools analyzed in duplicate. **a**-**e** Data were tested by one-way ANOVA. ns: not significant, *: *P* < 0.05, **: *P* < 0.01, ****: *P* < 0.0001 **f** Protein expression of phosphorylated NF-kB and phosphorylated H2A.X were detected by Western blotting. Shown are representative examples of two VSMC pools derived from 2 to 4 animals each. Upper panel: numbers represent the ratio of phospho-NF-kB to total NF-kB, normalized to GAPDH expression in the same sample. Lower panel: numbers represent the ratio of phospho-H2A.X and GAPDH. The expression in proliferating WT VSMC was set as 1.0

Because of its role as an inflammasome sensor in macrophages and other cell types, we next investigated whether inflammasome gene expression was affected by AIM2 in VSCM in vitro (Fig. 3 and supplementary figure S3). As expected, *Aim2* mRNA was not detectable in *Aim2−/−* VSMC (Fig. 3a). In addition, mRNA expressions of the inflammasome components *Nlrp3* and *Il1b* were significantly lower in *Aim2−/−* VSMC compared with WT VSMC in both, normal growth medium and after shifting the cells to mineralization medium for 2 weeks (Fig. 3b, c). Release of mature IL-1β from VSMC did not differ between the genotypes (Fig. 3d). In contrast, release of IL-6 was significantly lower in *Aim2−/−* VSMC (Fig. 3e). In line with the lower *Nlrp3* and *Il1b* transcription levels, expression of the inflammatory transcription factor phospho-NF-kB (Ser536) was reduced in *Aim2−/−* VSMC compared with WT VSMC, despite an increased DNA damage in these cells, as indicated by phosphorylation of Histone H2A.X at Ser139 (Fig. 3f). Together these data suggest a reduced inflammatory response in *Aim2−/−* VSMC.

### Aim2 deficiency reduces AA incidence and affects aortic remodeling in AngII-induced aortic aneurysm

**Fig. 4:**
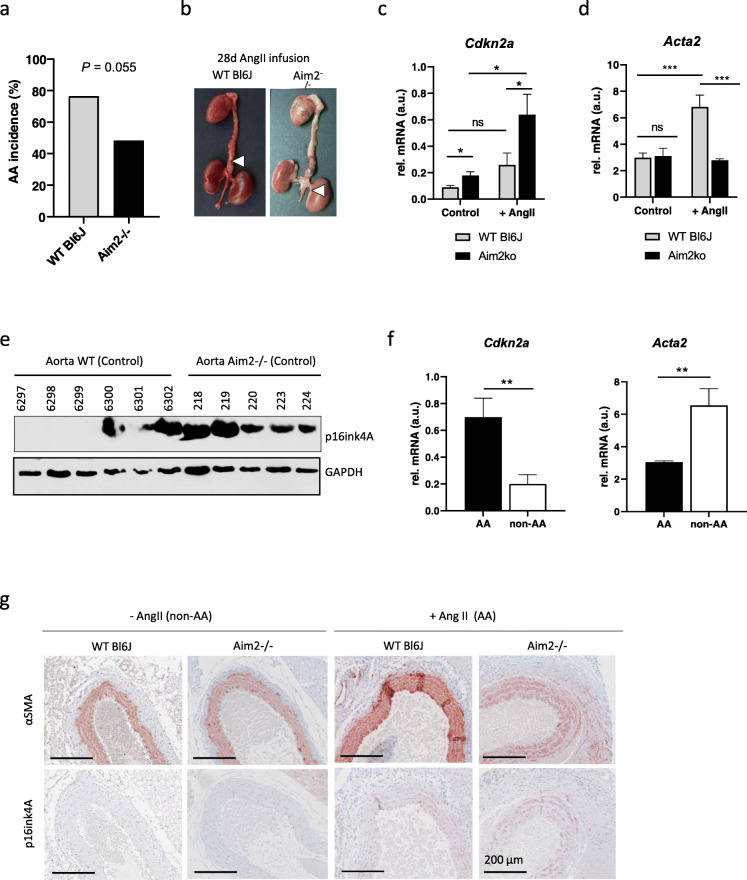
Involvement of Aim2 deficiency in AA formation. **a** Quantitative analysis of AA incidence in AngII–infused WT mice (*n* = 31) and *Aim2−/−* mice (*n* = 17). Data were analyzed by one-sided Fisher’s exact test. **b** Representative images of aortas from WT and *Aim2−/−* mice infused with 1500 ng/kg/min Ang II for 28 days. **c**, **d** Total mRNA was extracted from normal aortas or aortic aneurysms of untreated WT (*n* = 6), untreated *Aim2−/−* (*n* = 7), AngII-infused WT (*n* = 3) and AngII-infused *Aim2−/−* (*n* = 3) mice. **c** Expression of *Cdkn2a* mRNA is increased in aortas from *Aim2−/−* mice **d** *Acta 2* mRNA is increased in aortas from AngII infused WT mice. **e** Western blot, demonstrating increased expression of p16ink4A in aortic lysates of untreated *Aim2−/−* mice. **f** Increased mRNA expression of *Cdkn2a (p16)* and reduced mRNA expression of *Acta2* was detected in AA compared with non-AA from AngII infused animals. Bars show the mean ± SEM. **c**, **d**, **f** Data were tested by one-way ANOVA. ns: not significant, *: *P* < 0.05, **: *P* < 0.01, ***: *P* < 0.001. **g** Aortic sections immunohistochemically stained for α-SMA and p16ink4A

Considering the impact of *Aim2* deficiency on VSMC calcification and inflammatory response, we hypothesized that *Aim2* knockout might reduce AA formation in vivo. To investigate this hypothesis, we infused *Aim2−/−* and WT mice with AngII for 4 weeks. Four out of 31 (13%) *Aim2−/−* mice and four out of 17 (23%) WT mice died before the 28d infusion period following aortic rupture. Consistent with previous reports from AngII-infused *Apoe−/−* mice (Daugherty et al. 2000; Usui et al. 2015), aortic aneurysms were induced in 76% (13/17) of WT mice. In contrast, only 48% (15/31) of *Aim2−/−* mice developed an AA (Fig. 4a). Although the difference in AA incidence was not statistically significant (Fisher’s exact test, *P* = 0.055), we aimed to elucidate the aortas in more detail. Apparently, all aneurysms in WT mice were located either in the suprarenal or thoracic aorta. In contrast, in *Aim2*-deficient mice, all aneurysms were located in the infrarenal aorta (Fig. 4b). The reason for this difference is unclear and was not further analyzed.

Plasma levels of IL-1β and IL-6 did not significantly differ between WT and *Aim2−/−* mice after 28 days of AngII infusion (supplementary figure S4). Moreover, mRNA expression levels of the inflammatory cytokines *Il1b, Il6, Il18* and *Mcp1* were similar in aortas of AngII-infused WT and *Aim2−/−* mice (supplementary figure S4c), indicating that there was no difference in inflammation at the end of the experiments. In contrast, mRNA expression of the senescence marker *Cdkn2A/p16ink4A* was increased in *Aim2−/−* aortas from both, untreated and AngII infused mice (Fig. 4c), whereas Acta2 expression was only increased in aortas form AngII infused WT mice (Fig. 4d). Increased expression of p16ink4A protein was also detected by Western blotting of aortas derived from non-infused (control) *Aim2−/−* mice (Fig. 4e). When AA samples were compared with normal aortas, *Cdkn2A/p16ink4A* was elevated and *Acta2* was decreased in either genotype (Fig. 4f). Accordingly, protein expression of p16ink4A was significantly increased in AA samples, compared with normal aortas (Supplementary figure S5a, b).

**Fig. 5:**
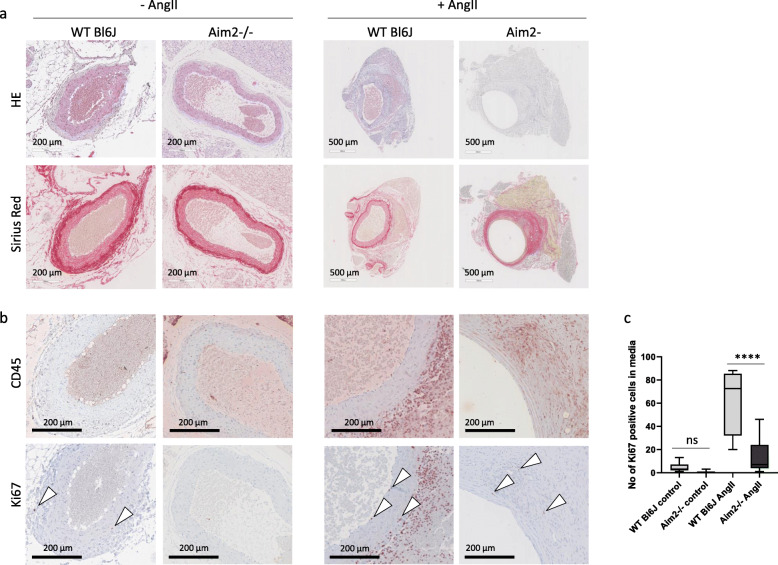
Representative histological aortic sections derived from untreated and AngII infused mice. **a** Samples were stained with hematoxylin/eosin (HE) for morphological analysis and with Sirius Red for detection of collagens. **b** Immunohistochemical analysis of leukocyte infiltration (CD45) and proliferating cells (Ki67); *n* = 3 for WT - Ang II; *n* = 6 for *Aim2−/−* − AngII; *n* = 4 for WT + Ang II; *n* = 6 for *Aim2−/−* + AngII. **c** Quantitative analysis of proliferating cells (Ki67 positive) within the media of abdominal aortic regions. Proliferating (Ki67 positive) cells were counted in abdominal aortic sections of both, dilated and undilated samples. Shown is the mean and SD of WT control (*n* = 7), *Aim2−/−* control (*n* = 6), WT + AngII (*n* = 4), and *Aim2−/−* + AngII (*n* = 11) samples. Data were analyzed. by one-way ANOVA. ns: not significant, ****: *P* < 0.0001

To investigate the role of VSMC in AA formation, we performed immunohistochemical analysis and found that p16ink4A was particularly expressed in VSMC (αSMA-positive cells) of AA samples and adjacent adventitial regions of WT and *Aim2−/−* mice, respectively, whereas it was completely absent in VSMC of healthy aortas (Fig. 4g). In line with the mRNA data, expression of αSMA was stronger in AA samples of AngII infused WT mice (Fig. 4g). The composition of AAs, as determined by hematoxylin and Sirius Red staining, did not differ significantly between the two genotypes (Fig. 5a). In addition, AAs of both genotypes were equally infiltrated with leukocytes, as indicated by CD45 expression (Fig. 5b). In contrast, the number of proliferating cells (Ki67 positive) was significantly lower in aortic medias from control and AngII infused *Aim2−/−* mice, compared with WT mice (Fig. 5b and c), which is in good agreement with the in vitro data, showing a lower viability and growth rate of *Aim2−/−* VSMC.

### Aim2 deficiency and inflammasome activation in VSMC derived from AngII-infused mice

To determine whether the VSMC phenotype was persistently affected by the 28-day-AngII-infusion, aortic VSMC were isolated from AngII-infused animals and grown in vitro. Similar to control animals, VSMC derived from AngII treated *Aim2−/−* animals were larger and grew more slowly than VSMC derived from AngII treated WT animals (supplementary Figure S5c). In contrast to VSMC from untreated mice, mRNA expression of the innate immunity genes *Nlrp3 and Il1b* did not differ between *Aim2* deficient and WT VSMC of AngII infused mice. Moreover, mRNA expression of *Casp1, Asc/Pycard,* and *Il18* was similar in both genotypes (supplementary Figure S5d).

## Discussion

The primary novel finding of this study is that AIM2 is functionally involved in phenotype transition of VSMC. *Aim2* deficiency resulted in reduced proliferation, accelerated senescence and an early shift to a synthetic phenotype of murine aortic VSMC. This was accompanied by increased calcification but reduced inflammatory response of *Aim2−/−* VSMC. In line with the reduced inflammation, the AA incidence was lower in *Aim2* knockout mice in the in vivo model of AngII-infused mice. At first sight, the reduced in vitro proliferation rate in combination with a synthetic phenotype of *Aim2* deficient VSMC appears to contradict previous reports, which showed that proliferating activity is higher in synthetic than in contractile VSMC. However, one should bear in mind that the cells have been growing in vivo for 6 months before they were isolated for in vitro culturing. Potentially, *Aim2* deficient VSMC displayed faster proliferation in vivo and are therefore already closer to replicative senescence than their WT counterparts. Although we have not determined the in vivo proliferation rate, this might explain the higher p16ink4A expression along with earlier senescence that was observed here reproducibly in all *Aim2−/−* VSMC cultures analyzed. Moreover, in contrast to WT VSMC, Acta2/αSMA expression was not reduced in *Aim2* deficient VSMC, when the cells reached senescence. Together with the reduced inflammatory activity of *Aim2−/−* VSMC, this agrees with a protective mechanism of *Aim2* deficiency in aortic VSMC.

The mechanism by which *Aim2* deficiency results in accelerated senescence and calcification of VSMC is currently unknown. AIM2 is a dsDNA sensor that is mainly activated in monocytes and macrophages by cytoplasmic DNA, derived from pathogens or neighboring necrotic cells (Briard et al. 2020). In addition, there is accumulating evidence for AIM2 to be involved in sustaining mitochondrial function by detecting mitochondrial DNA (mtDNA) in various human diseases, including cancer and diabetes type 2 (Bae et al. 2019; Qi et al. 2020), as well as in detecting virus-induced oxidized DNA (Moriyama et al. 2020). In neurodevelopment, AIM2 was recently shown to be involved in surveillance of DNA damage of neurons (Lammert et al. 2020). Finally, mitochondrial ROS (reactive oxygen species) overproduction and mtDNA damage are causally related to age-related vascular dysfunction in humans (Csiszar et al. 2009; Mikhed et al. 2015). Although we have not analyzed DNA damage and AIM2 response here, this suggests that AIM2-mediated DNA surveillance might be important for VSMC function and AAA formation, as well. It is tempting to speculate that oxidative stress, resulting in mitochondrial damage and mtDNA release into the cytoplasm of VSMC, might be detected by AIM2, which is necessary for DNA damage response. Deletion or mutation of AIM2 might therefore result in increased DNA-damage-induced VSMC senescence and/or osteoblast-like differentiation. Interestingly, and according to our animal experiments, this in turn resulted in a lower responsiveness to AngII and thus a lower AA incidence than in WT mice. It should be noted here, that the most commonly used WT mouse strain C57Bl/6 J and the mutated *Aim2−/−* strain are both affected by deficiency of mitochondrial Nicotinamide Nucleotide Transhydrogenase (NNT), a core protein in the mitochondrial respiratory chain, resulting in redox abnormalities, which are not present in the C57Bl/6 N WT mouse strain (Simon et al. 2013). In a recent study, that was performed in parallel to the experiments presented here, we could show that the C57Bl/6 J, NNT deficient mouse strain is particularly susceptible to AngII-induced aneurysm formation, and VSMC derived from C57BL/6 J mice display increased oxidative stress and DNA damage (Wortmann et al., unpublished data). Potentially, *Aim2* deficiency in the NNT deficient background might result in different phenotypic changes of VSMC that in NNT-proficient murine and human VSMC. Alternatively, the protective effect of *Aim2* deficiency on AA formation that was observed here, is based on macrophages, which have not been investigated in this study. It will thus be important to further study AIM2 and its role in different cell types during AA formation in both, mice and humans.

Regardless of the mechanism, our findings are in line with recent studies demonstrating that inflammasome activation leads to the development of AngII-induced aortic aneurysm in hypercholesterolaemic (*Apoe−/−*) mice (Usui et al. 2015). According to this previous study, AA was formed in 10/14 (~ 71%) of *Apoe−/−* mice, whereas only 2/8 (25%) of *Apoe−/− Nlrp3−/−* mice and 4/14 (~ 29%) *Apoe−/− Casp1−/−* mice developed an AA after AngII infusion. Using WT instead of *Apoe−/−* mice as a background, we here found AAs in 13/17 (76%) of WT mice, but only in 15/31 (48%) of *Aim2−/−* mice. AIM2 may thus be added to the list of inflammasome components, interfering with AA formation, although the extent of this interference appears to be weaker for AIM2 than for NLRP3 or Caspase-1. Whether the here observed effects of *Aim2*-deletion will be stronger pronounced on the background of *Apoe−/−* mice, is currently under investigation.

Increasing evidence suggests that the innate immune system and in particular inflammasomes, are major contributors to cardiovascular disease, including AA formation (Dihlmann et al. 2014; Johnston et al. 2013, 2014; Li et al. 2014; Wortmann et al. 2019a, b). However, it is unclear so far, how the innate immune system contributes to AA formation and which cell types are the major players in disease progression. Inflammasome activation has been suggested to be involved in VSMC phenotype transition during vascular remodeling (Gardner et al. 2015; Ren et al. 2017; Sun et al. 2017; Wen et al. 2013; Wu et al. 2017). Whereas these studies focused predominantly on the NLRP3 inflammasome, we here present evidence for a role of AIM2 in regulating the VSMC response to AA-inducing mechanisms. According to our data, *Aim2* deletion resulted in reduced inflammatory response of VSMC from control animals. However, *Aim2* deletion did not affect inflammatory cell infiltration of AAs after 28 days of continuous AngII infusion. *Aim2* deficiency might thus exert different impact on VSMC and myeloid cells. As mentioned above, we cannot exclude that the protective effect of AIM2 might be independent from reducing the inflammatory response of VSMC, i.e. resulting from some so far unknown mechanism. No robust differences in elastin breaks or its organization were apparent in histological samples. Because of the limited number of histologically analyzable aneurysm samples, we did not examine Mmp expression in histological samples for statistical analysis. Given that *Mmp9* mRNA expression in VSMC was not associated with the genotype, we assume that elastin degradation is not affected by Aim2−/− deletion.

Analysis of calcification-associated mRNA expression suggested that *Aim2* deletion results in persistent induction of *Bmp4* and repression of *Tnfsf11/Rankl* in murine VSMC. BMP4 is a member of the bone morphogenetic protein family and a marker for osteochondrogenic differentiation in bone, and other tissues (Carreira et al. 2014). In contrast, *Tnfsf11/Rankl* is considered an osteoclast differentiation factor, (Karsenty 1999). Recently, it was demonstrated that human and murine aneurysms express high levels of *Tnfsf11/Rankl* and are more closely associated with the osteoclast-like catabolic degradation of the aorta than with the osteoblast-like anabolic processes of arterial calcification (Kelly et al. 2019; Takei et al. 2016). In addition, RANKL was shown to mediate osteoclastogenic differentiation of macrophages in the abdominal aorta of AngII-infused *Apoe* deficient mice (Tanaka et al. 2018). Correspondingly, the data presented here suggest that *Aim2* deficiency might trigger VSMC to a more osteoblast-like phenotype transition by reducing expression of *Tnfsf11/Rankl* and inducing expression of *Bmp4*. Conversely, *Aim2* expression in WT VSMC, i.e. in response to cytosolic DNA, appears to allow for an osteoclast-like differentiation of VSMC, thereby promoting catabolic degradation. Thus, shifting of VSMC to a phenotype predisposed for anabolic calcification, with increased senescence and reduced proliferation does not necessarily weaken the aortic wall. This conclusion is further supported by previous studies demonstrating that vessels with greater calcification exhibit reduced aneurysm growth and the areas of aneurysmal vessels with less calcification may be the most likely sites of rupture (Lindholt 2008; Nakayama et al. 2016; Raghavan et al. 2006). In line with the reduced *Rankl/*sRANKL levels, expression of the osteogenic transcription factor *Runx2* was reduced in aging and calcifying *Aim2−/−* VSMC compared with WT VSMC. RUNX2 has been demonstrated to directly bind to and control the *Rankl* promoter, thereby regulating its expression (Byon et al. 2011). It should be noted here, that the decrease of an osteoclast marker does not necessarily provide evidence for osteogenic differentiation or the lack thereof. It was demonstrated that calcification may likewise occur without osteogenic differentiation (O’Neill and Adams 2014). Moreover, our study is limited by lacking analysis of additional osteogenic differentiation markers such as osteocalcin, osteopontin, osteoportegerin, bone sialoprotein, or MGP. Thus, the precise role of AIM2 in regulating osteogenic signaling in VSMC remains to be determined.

## Conclusion

In summary, our data demonstrate that AIM2 affects several molecular mechanisms involved in aortic remodeling and murine AA formation. The signaling pathways resulting in reduced proliferation and increased senescence of *Aim2* deficient VSMC remain to be investigated. The mechanism resulting in increased calcification of *Aim2* deficient VSMC has been elucidated in part. Based on our data, we suggest the following model (Table 1): In *Aim2*-deficient VSMC, the absence of the osteoclast differentiation factor *Tnfsf11/Rankl,* paralleled by high *Bmp4* levels, triggers the cells to differentiate into osteoblast-like cells with a low inflammatory response. In contrast, WT VSMC, expressing high *Tnfsf11/Rankl* and low *Bmp4 levels* differentiate into osteoclast-like VSMC with a high inflammatory response. Whether AIM2 deficiency results in similar VSMC phenotype transitions in human AAA, and whether this is associated with AAA pathophysiology, needs to be investigated, i.e. by studying AIM2 mutations/expression particularly in calcified versus non-calcified AAA tissue samples.

**Table 1:** Effects of Aim2 expression/knockout on VSMC differentiation and aortic remodeling

VSMC Genotype	Aim2 −/−	WT
*Rankl/Tnfsf11* expression	low	high
*Bmp4* expression	high	low
Calcification in mineralization medium (OM)	high	low
Osteochondrogenic differentiation	Osteoblast-like	Osteoclast-like
Role in AAA	Anabolic calcification; reduced incidence of aortic dilation	Catabolic degradation; high incidence of aortic dilation
*Nlrp3* expression	low	high
*Il1b* expression	low	high
NF-κB (p65) activity	low	high
Il-6 release	low	high
Inflammatory response	low	high

## Supplementary information

**Additional file 1: Supplementary table 1.** primer sequences for real-time PCR. **Supplementary figure S1.** Mouse VSMC (C57Bl/6 N) were grown for 27 days in Smooth muscle cell growth medium or in osteoblast mineralization medium. Subsequently calcification was visualized in both cultures by staining with Alizarin Red S as described in Materials and methods. Images were taken using a light microscope with phase contrast (a) or bright field (b). **Supplementary figure S2.** Analysis of mRNA expression by RT-qPCR. Total RNA was extracted from VSMC grown in vitro with normal growth medium (−OM) or osteoblast mineralization medium (+ OM). A.u. arbitrary unit; relative expression of target gene was normalized against expression of housekeeping genes (*Actb, Gapdh* and *B2m*). Bars show the mean +/− SD of *n* = 3 measurements of 3 VSMC pools, each. Data were statistically analyzed by one-way-ANOVA. *: *P* < 0.05; **: *P* < 0.01; ****: *P* < 0.0001; ns: not significant. **Supplementary figure S3.** Expression of genes encoding inflammsome components. Analysis of mRNA expression of Asc (a), and Casp1 (b) by real-time reverse-transcriptase-polymerse chain reaction (*n* = 3 measurements of 3 experiments for each). Total RNA was extracted from VSMC grown in vitro with normal growth medium (−OM) or osteoblast mineralization medium (+ OM). A.u. arbitrary unit; relative expression of target gene was normalized against expression of housekeeping genes (*Actb, Gapdh* and *B2m*). *Asc* expression was similarly low in VSMC of both genotypes grown in normal growth medium. After shifting the cells to mineralization medium, *Asc* mRNA levels were significantly increased in both genotypes (−OM versus + OM: *P* < 0.001), although the increase was less intense in *Aim2−/−* VSMC (a). In contrast, expression of *Casp1* was increased in *Aim2−/−* compared with WT VSMC in either growth medium (b). Data were statistically analyzed by one-way-ANOVA. *: *P* < 0.05; **: *P* < 0.01; ****: *P* < 0.0001; ns: not significant. **Supplementary figure S4.** Plasma levels of inflammatory cytokines IL-1β (a) and IL-6 (b) were determined by specific ELISAs from AngII-treated WT Bl6J and *Aim2* deficient mice at the end of the 28d infusion. Data show the mean and standard deviations of *n* = 11 mice for each group. c. Real-time RT-PCR of aortas showing *Il1b, Il6, Il18 and Mcp1* gene expression of AngII-infused WT Bl6J (*n* = 3) and AngII-infused *Aim2−/−* (*n* = 3) mice. Data show the mean +/− SEM of three mice analyzed in triplicates. n.s.: not significant. **Supplementary figure S5.** a. Protein Expression of the senescence marker p16ink4A is increased in AA. Mouse aortas with and without aneurysms (AAA) were lysed and analyzed by Western blotting. b. Quantitative analysis of p16ink4A expression normalized to Vinculin. Blots were scanned and signals were densitometrically analyzed by Image J software. c. Mouse VSMC (C57Bl/6 J) were isolated from AngII treated mice and grown in vitro. Each pool contained VSMC of 3 animals. VSMC derived from *Aim2−/−* mice are larger, rounded and contain big vacuoles. The picture shows representative cultures of passage 3 from *n* = 3 (*Aim2−/−)* and *n* = 3 (WT) VSMC pools. d. mRNA levels of VSMC derived from AngII infused WT Bl6J (*n* = 3 pools) and *Aim2−/−* (*n* = 3 pools) animals.). A.u. arbitrary unit; relative expression of target gene was normalized against expression of housekeeping genes (*Actb, Gapdh* and *B2m*).

## Data Availability

All relevant data supporting the conclusions of this article is included within the article and its additional files. Original raw data of real-time RT-PCR and original colorimetric data from the ELISAs are available upon request.

## Abbreviations

AA: Aortic aneurysm
AAA: Abdominal aortic aneurysm
ACTB: Actin B
AIM2: Absent in Melanoma 2
AngII: Angiotensin II
APC: Apoptosis-associated speck-like protein containing a caspase recruitment domain
Apoe: Apolipoprotein E
ARS: Alizarin Red S
αSMA: Alpha-smooth muscle actin
B2M: Beta-2-microglobulin
BMP: Bone morphogenic protein
CASP1: Caspase-1
CDKN2A: Cyclin dependent kinase inhibitor 2A
ECL: Enhanced chemoluminescence
GAPDH: Glyceraldehyde-3-phosphate dehydrogenase
IL: Interleukin
MCP1: Monocyte chemoattractant protein 1
NLRP3: NLR family pyrin domain containing 3
PBS: Phosphate buffered saline
qRT-PCR: Quantitative reverse transcription polymerase chain reaction
RANKL: Receptor Activator of NF-kB (nuclear factor ‘kappa-light-chain-enhancer’ of activated B-cells) ligand
RUNX2: Runt-related transcription factor 2
SOX9: SRY-related HMG-(high mobility group) box gene 9
TNFSF11: Tumor necrosis factor superfamily member 11
VSMC: Vascular smooth muscle cell
WT: Wild type
