# Supplementary material for: Deficiency in Aim2 affects viability and calcification of vascular smooth muscle cells from murine aortas and angiotensin-II induced aortic aneurysms
Source: Mol Med. 2020 Sep 15;26:87. doi: 10.1186/s10020-020-00212-z (PMC7493160; doi:10.1186/s10020-020-00212-z)
Supplement: Supplementary file 1 — Additional file 1: Supplementary table 1. primer sequences for real-time PCR. Supplementary figure S1. Mouse VSMC (C57Bl/6 N) were grown for 27 days in Smooth muscle cell growth medium or in osteoblast mineralization medium. Subsequently calcification was visualized in both cultures by staining with Alizarin Red S as described in Materials and methods. Images were taken using a light microscope with phase contrast (a) or bright field (b). Supplementary figure S2. Analysis of mRNA expression by RT-qPCR. Total RNA was extracted from VSMC grown in vitro with normal growth medium (−OM) or osteoblast mineralization medium (+ OM). A.u. arbitrary unit; relative expression of target gene was normalized against expression of housekeeping genes (Actb, Gapdh and B2m). Bars show the mean +/− SD of n = 3 measurements of 3 VSMC pools, each. Data were statistically analyzed by one-way-ANOVA. *: P < 0.05; **: P < 0.01; ****: P < 0.0001; ns: not significant. Supplementary figure S3. Expression of genes encoding inflammsome components. Analysis of mRNA expression of Asc (a), and Casp1 (b) by real-time reverse-transcriptase-polymerse chain reaction (n = 3 measurements of 3 experiments for each). Total RNA was extracted from VSMC grown in vitro with normal growth medium (−OM) or osteoblast mineralization medium (+ OM). A.u. arbitrary unit; relative expression of target gene was normalized against expression of housekeeping genes (Actb, Gapdh and B2m). Asc expression was similarly low in VSMC of both genotypes grown in normal growth medium. After shifting the cells to mineralization medium, Asc mRNA levels were significantly increased in both genotypes (−OM versus + OM: P < 0.001), although the increase was less intense in Aim2−/− VSMC (a). In contrast, expression of Casp1 was increased in Aim2−/− compared with WT VSMC in either growth medium (b). Data were statistically analyzed by one-way-ANOVA. *: P < 0.05; **: P < 0.01; ****: P < 0.0001; ns: not significant. Supplementary figure S4. [file 10020_2020_212_MOESM1_ESM.pptx]

## Slide 1
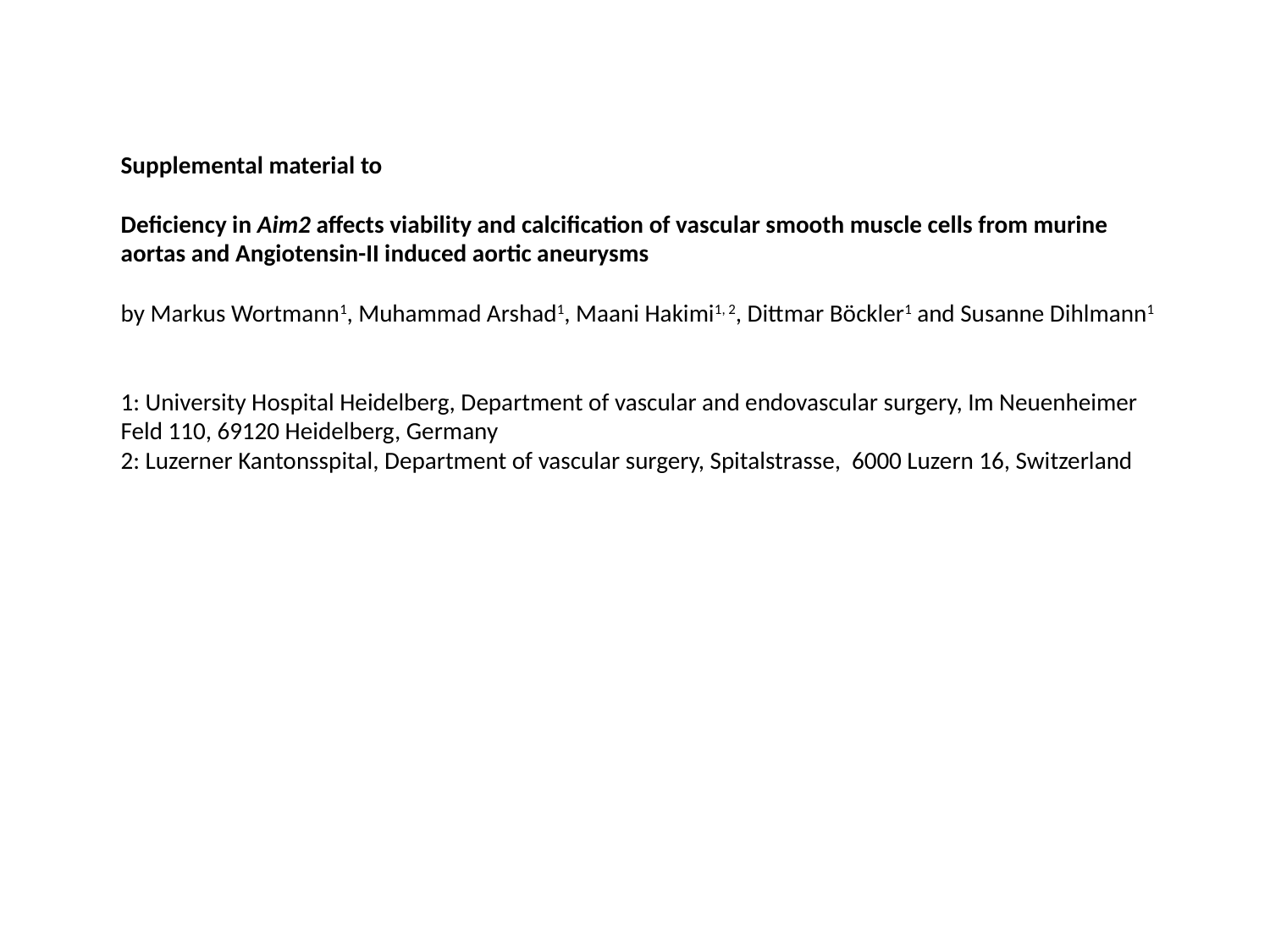

Supplemental material to
Deficiency in Aim2 affects viability and calcification of vascular smooth muscle cells from murine aortas and Angiotensin-II induced aortic aneurysms
by Markus Wortmann1, Muhammad Arshad1, Maani Hakimi1, 2, Dittmar Böckler1 and Susanne Dihlmann1
1: University Hospital Heidelberg, Department of vascular and endovascular surgery, Im Neuenheimer Feld 110, 69120 Heidelberg, Germany
2: Luzerner Kantonsspital, Department of vascular surgery, Spitalstrasse, 6000 Luzern 16, Switzerland

## Slide 2
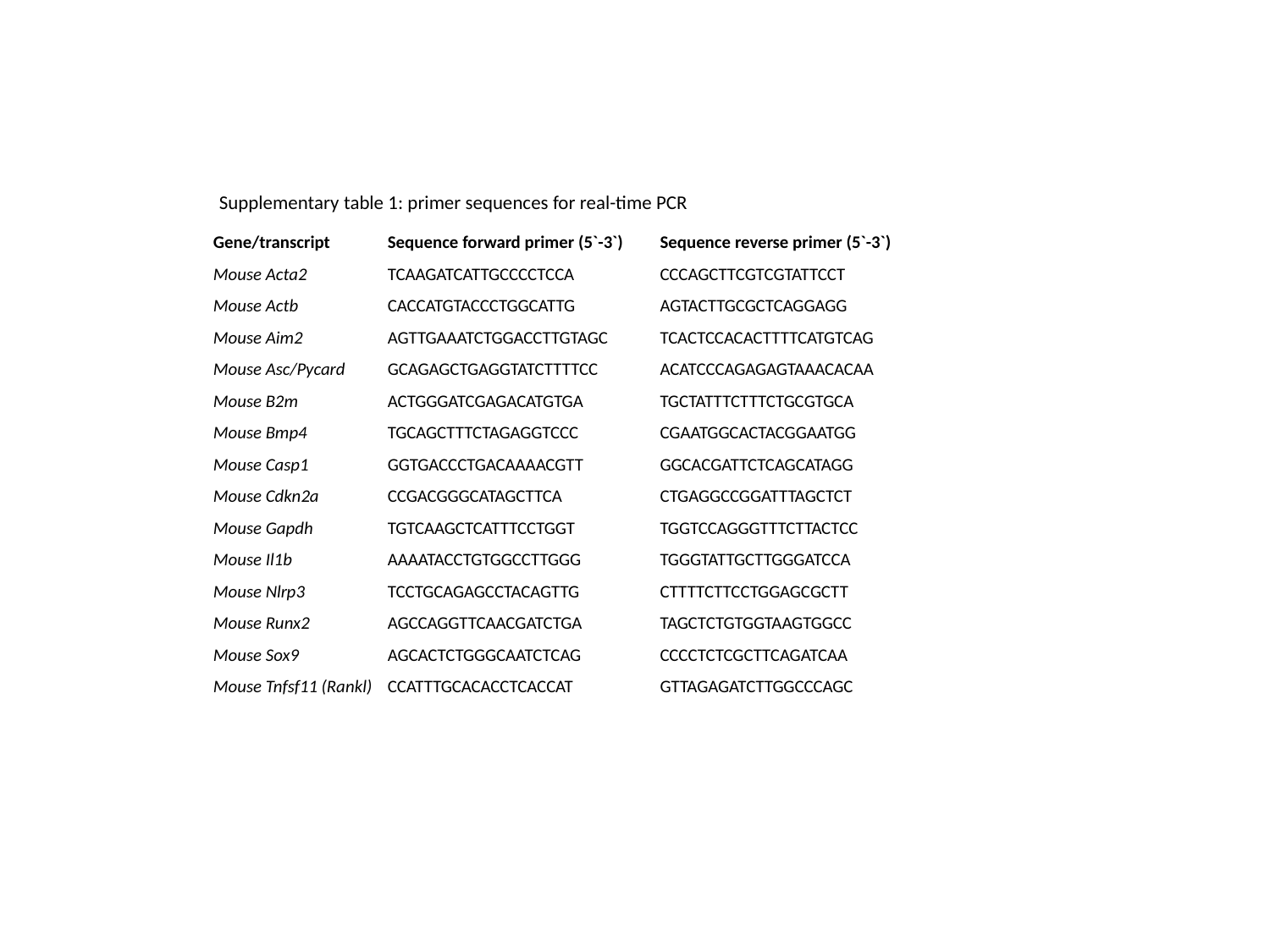

Supplementary table 1: primer sequences for real-time PCR
| Gene/transcript | Sequence forward primer (5`-3`) | Sequence reverse primer (5`-3`) |
| --- | --- | --- |
| Mouse Acta2 | TCAAGATCATTGCCCCTCCA | CCCAGCTTCGTCGTATTCCT |
| Mouse Actb | CACCATGTACCCTGGCATTG | AGTACTTGCGCTCAGGAGG |
| Mouse Aim2 | AGTTGAAATCTGGACCTTGTAGC | TCACTCCACACTTTTCATGTCAG |
| Mouse Asc/Pycard | GCAGAGCTGAGGTATCTTTTCC | ACATCCCAGAGAGTAAACACAA |
| Mouse B2m | ACTGGGATCGAGACATGTGA | TGCTATTTCTTTCTGCGTGCA |
| Mouse Bmp4 | TGCAGCTTTCTAGAGGTCCC | CGAATGGCACTACGGAATGG |
| Mouse Casp1 | GGTGACCCTGACAAAACGTT | GGCACGATTCTCAGCATAGG |
| Mouse Cdkn2a | CCGACGGGCATAGCTTCA | CTGAGGCCGGATTTAGCTCT |
| Mouse Gapdh | TGTCAAGCTCATTTCCTGGT | TGGTCCAGGGTTTCTTACTCC |
| Mouse Il1b | AAAATACCTGTGGCCTTGGG | TGGGTATTGCTTGGGATCCA |
| Mouse Nlrp3 | TCCTGCAGAGCCTACAGTTG | CTTTTCTTCCTGGAGCGCTT |
| Mouse Runx2 | AGCCAGGTTCAACGATCTGA | TAGCTCTGTGGTAAGTGGCC |
| Mouse Sox9 | AGCACTCTGGGCAATCTCAG | CCCCTCTCGCTTCAGATCAA |
| Mouse Tnfsf11 (Rankl) | CCATTTGCACACCTCACCAT | GTTAGAGATCTTGGCCCAGC |

## Slide 3
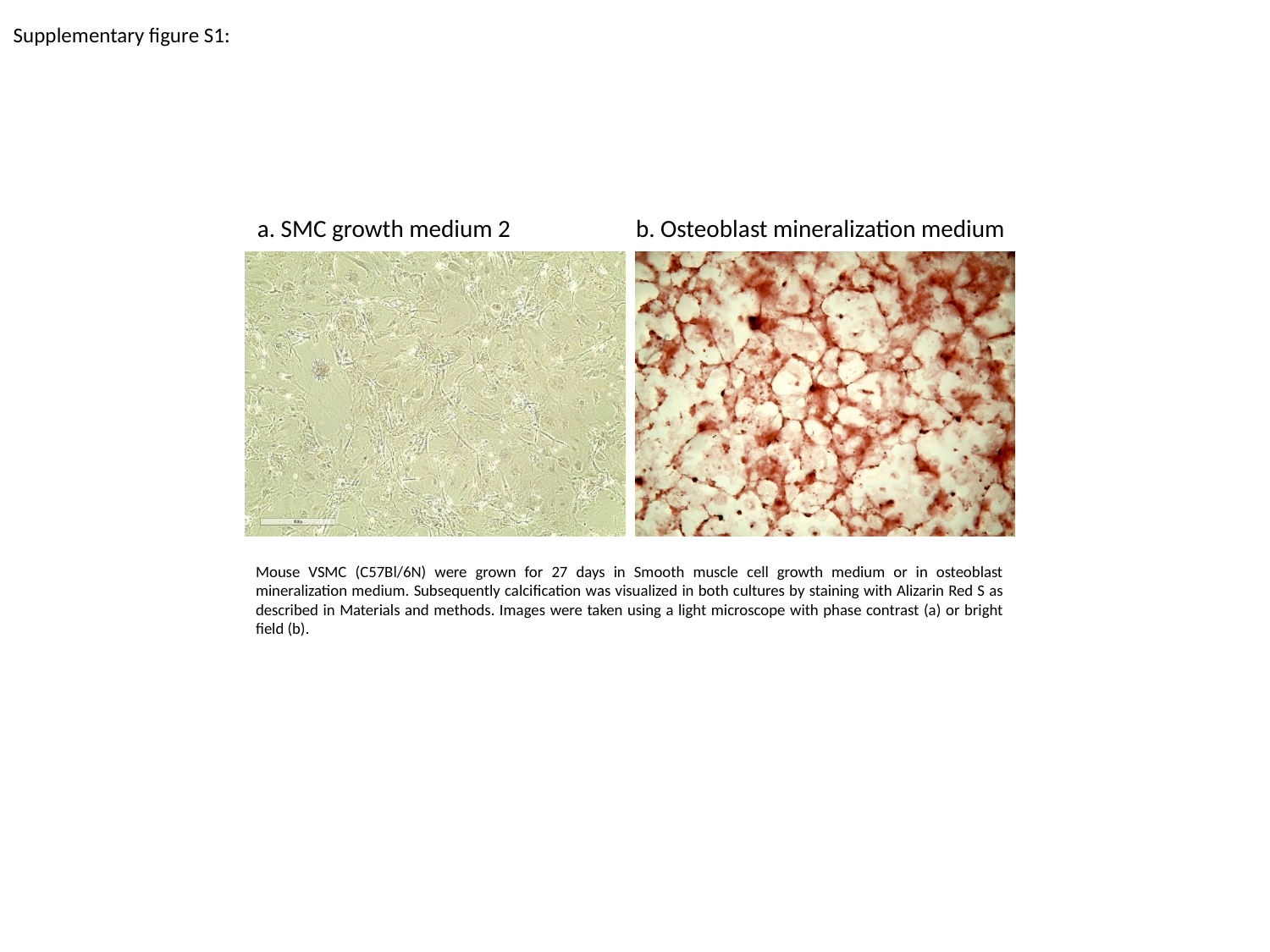

Supplementary figure S1:
a. SMC growth medium 2
b. Osteoblast mineralization medium
Mouse VSMC (C57Bl/6N) were grown for 27 days in Smooth muscle cell growth medium or in osteoblast mineralization medium. Subsequently calcification was visualized in both cultures by staining with Alizarin Red S as described in Materials and methods. Images were taken using a light microscope with phase contrast (a) or bright field (b).

## Slide 4
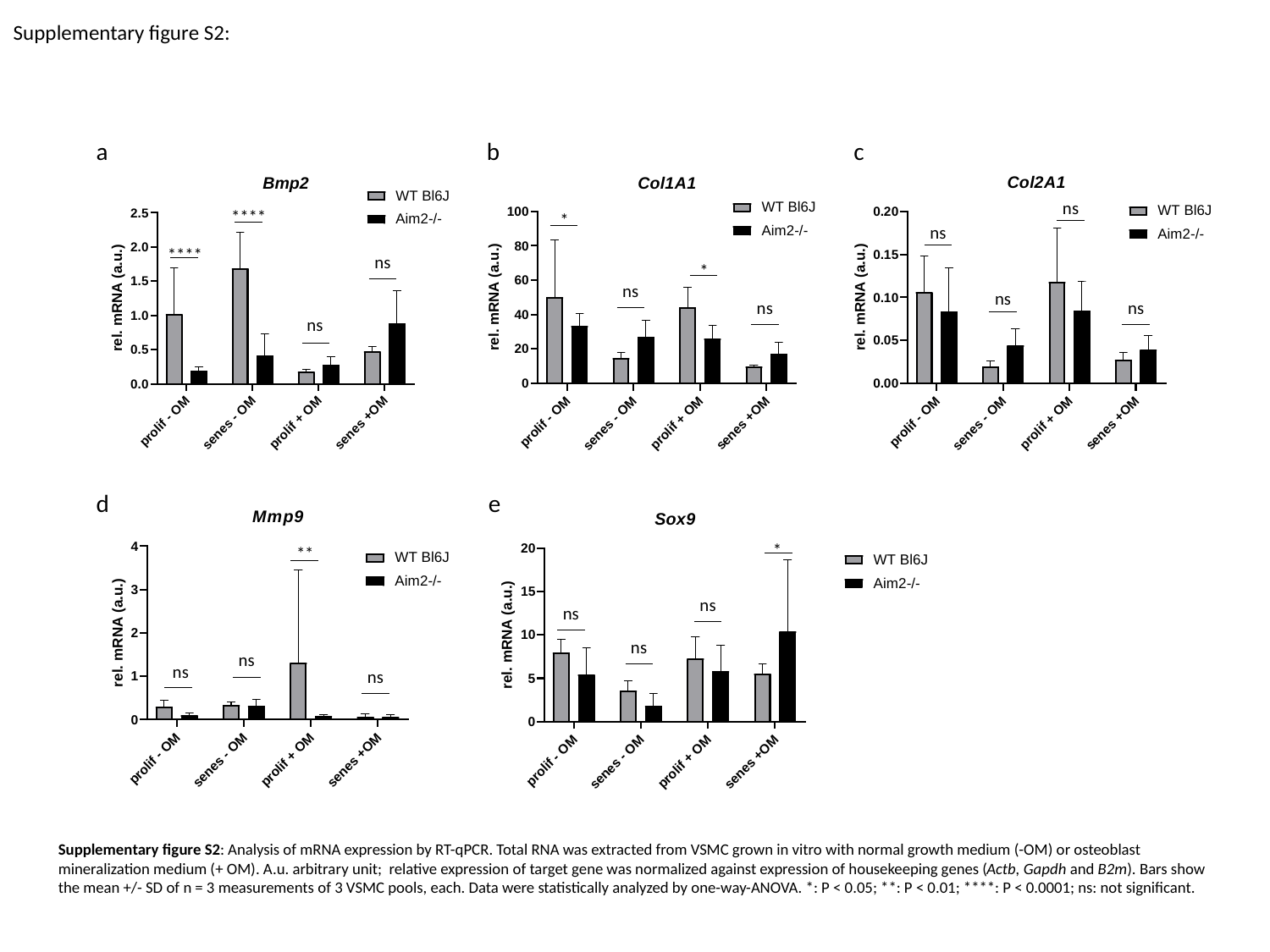

Supplementary figure S2:
a
b
c
ns
****
*
ns
****
ns
*
ns
ns
ns
ns
ns
d
e
*
**
ns
ns
ns
ns
ns
ns
Supplementary figure S2: Analysis of mRNA expression by RT-qPCR. Total RNA was extracted from VSMC grown in vitro with normal growth medium (-OM) or osteoblast mineralization medium (+ OM). A.u. arbitrary unit; relative expression of target gene was normalized against expression of housekeeping genes (Actb, Gapdh and B2m). Bars show the mean +/- SD of n = 3 measurements of 3 VSMC pools, each. Data were statistically analyzed by one-way-ANOVA. *: P < 0.05; **: P < 0.01; ****: P < 0.0001; ns: not significant.

## Slide 5
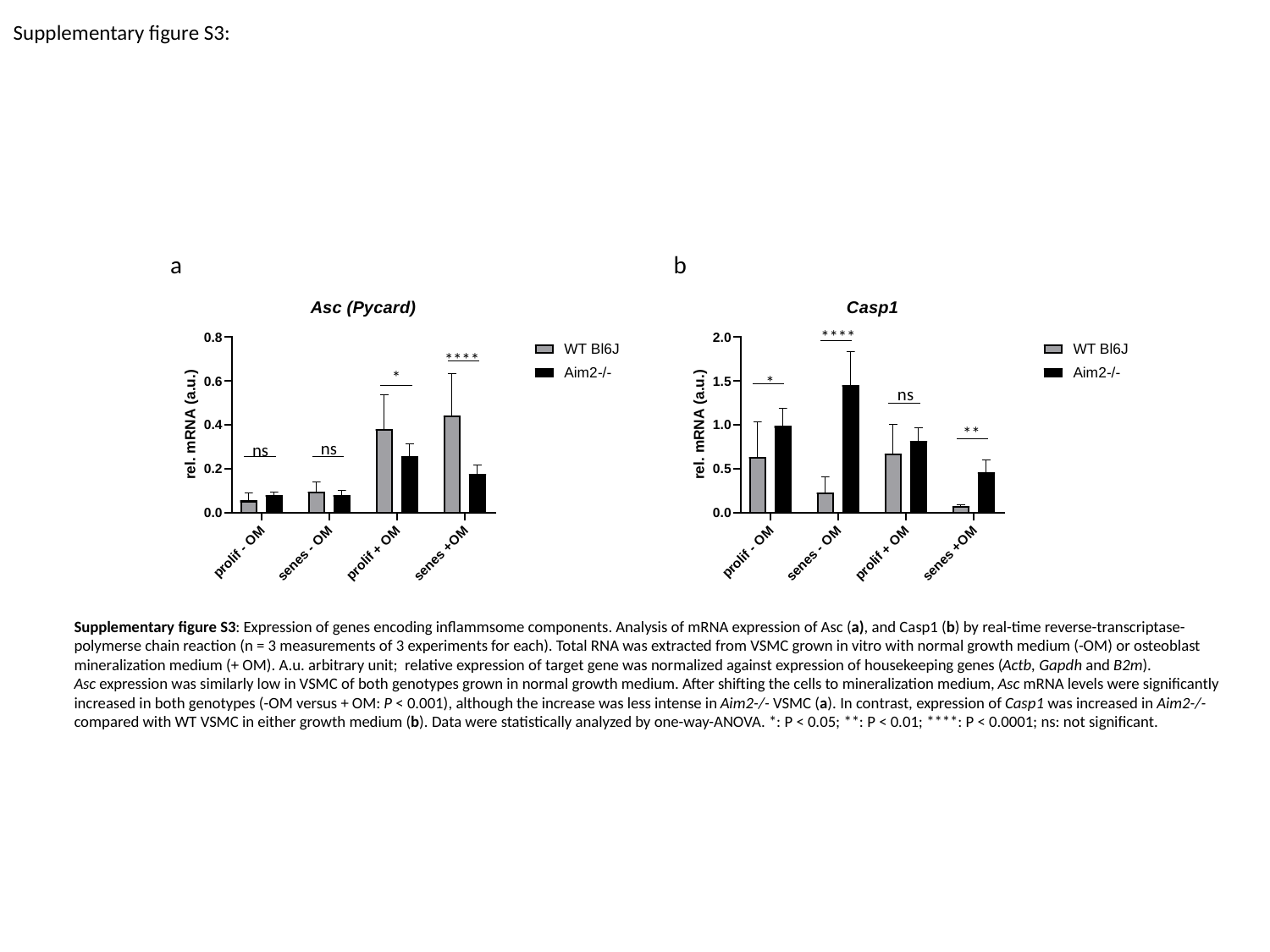

Supplementary figure S3:
a
b
****
****
*
*
ns
**
ns
ns
Supplementary figure S3: Expression of genes encoding inflammsome components. Analysis of mRNA expression of Asc (a), and Casp1 (b) by real-time reverse-transcriptase-polymerse chain reaction (n = 3 measurements of 3 experiments for each). Total RNA was extracted from VSMC grown in vitro with normal growth medium (-OM) or osteoblast mineralization medium (+ OM). A.u. arbitrary unit; relative expression of target gene was normalized against expression of housekeeping genes (Actb, Gapdh and B2m).
Asc expression was similarly low in VSMC of both genotypes grown in normal growth medium. After shifting the cells to mineralization medium, Asc mRNA levels were significantly increased in both genotypes (-OM versus + OM: P < 0.001), although the increase was less intense in Aim2-/- VSMC (a). In contrast, expression of Casp1 was increased in Aim2-/- compared with WT VSMC in either growth medium (b). Data were statistically analyzed by one-way-ANOVA. *: P < 0.05; **: P < 0.01; ****: P < 0.0001; ns: not significant.

## Slide 6
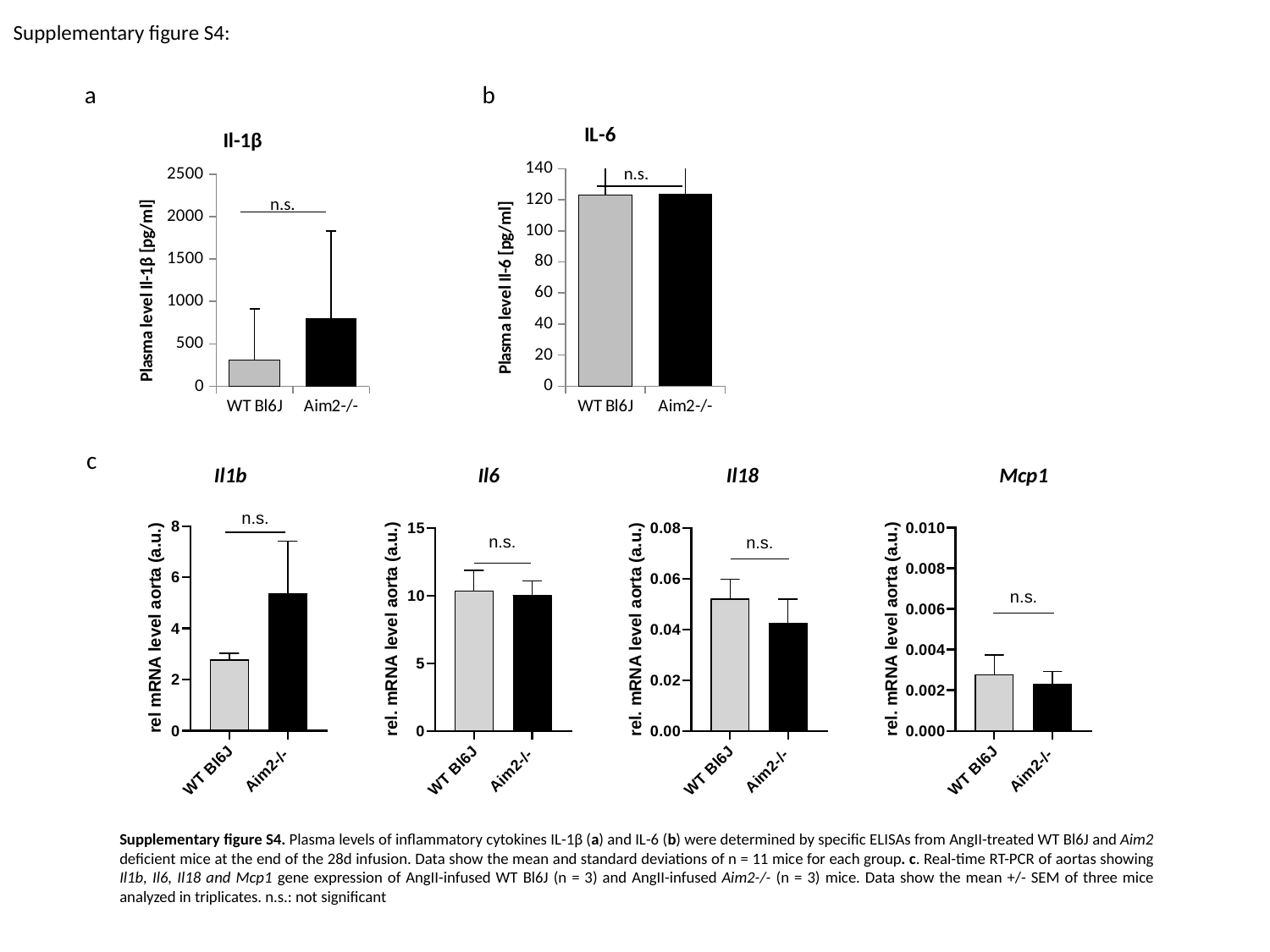

Supplementary figure S4:
a
b
### Chart: IL-6
| Category | mean |
|---|---|
| WT Bl6J | 122.9545454545455 |
| Aim2-/- | 124.00000000000004 |
### Chart: Il-1β
| Category | Mean |
|---|---|
| WT Bl6J | 309.35064935064946 |
| Aim2-/- | 806.7532467532469 |n.s.
n.s.
c
Il1b
Il6
Il18
Mcp1
Supplementary figure S4. Plasma levels of inflammatory cytokines IL-1β (a) and IL-6 (b) were determined by specific ELISAs from AngII-treated WT Bl6J and Aim2 deficient mice at the end of the 28d infusion. Data show the mean and standard deviations of n = 11 mice for each group. c. Real-time RT-PCR of aortas showing Il1b, Il6, Il18 and Mcp1 gene expression of AngII-infused WT Bl6J (n = 3) and AngII-infused Aim2-/- (n = 3) mice. Data show the mean +/- SEM of three mice analyzed in triplicates. n.s.: not significant

## Slide 7
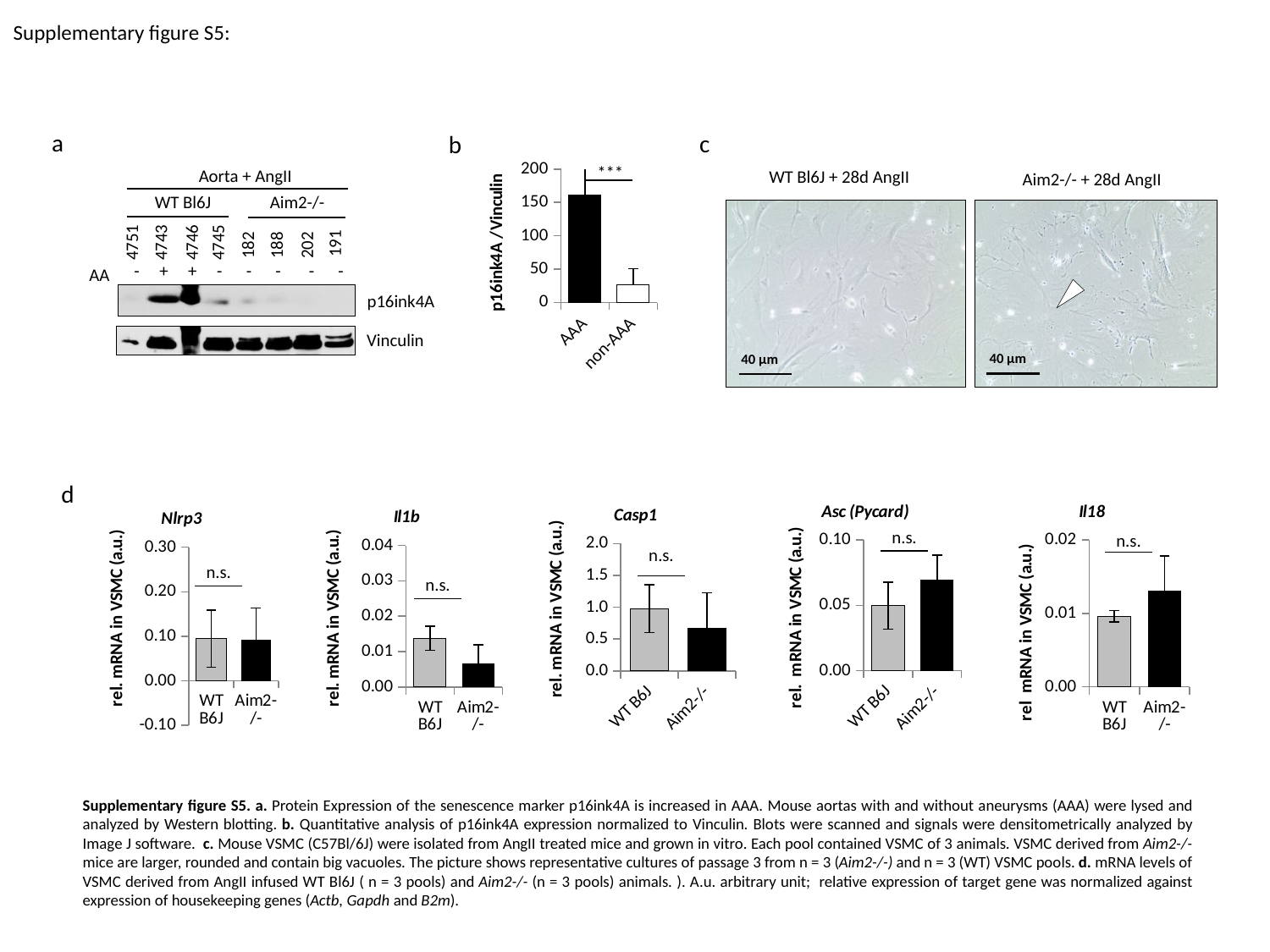

Supplementary figure S5:
a
c
b
***
### Chart
| Category | p16ink4A |
|---|---|
| AAA | 160.4370640652383 |
| non-AAA | 26.12492442041609 |Aorta + AngII
Aim2-/-
WT Bl6J
4751
4743
4746
4745
191
182
188
202
- + + - - - - -
AA
p16ink4A
Vinculin
WT Bl6J + 28d AngII
Aim2-/- + 28d AngII
40 µm
40 µm
d
### Chart: Asc (Pycard)
| Category | wo OM |
|---|---|
| WT B6J | 0.04960452243554502 |
| Aim2-/- | 0.06964259001551765 |
### Chart: Il18
| Category | wo OM |
|---|---|
| WT B6J | 0.00960340321660022 |
| Aim2-/- | 0.013055823064769753 |
### Chart: Casp1
| Category | wo OM |
|---|---|
| WT B6J | 0.9787974816772159 |
| Aim2-/- | 0.6797077011115554 |
### Chart: Il1b
| Category | wo OM |
|---|---|
| WT B6J | 0.013758371604939715 |
| Aim2-/- | 0.006747395560881566 |
### Chart: Nlrp3
| Category | wo OM |
|---|---|
| WT B6J | 0.09472042928345457 |
| Aim2-/- | 0.09294342587053028 |n.s.
n.s.
n.s.
n.s.
n.s.
Supplementary figure S5. a. Protein Expression of the senescence marker p16ink4A is increased in AAA. Mouse aortas with and without aneurysms (AAA) were lysed and analyzed by Western blotting. b. Quantitative analysis of p16ink4A expression normalized to Vinculin. Blots were scanned and signals were densitometrically analyzed by Image J software. c. Mouse VSMC (C57Bl/6J) were isolated from AngII treated mice and grown in vitro. Each pool contained VSMC of 3 animals. VSMC derived from Aim2-/- mice are larger, rounded and contain big vacuoles. The picture shows representative cultures of passage 3 from n = 3 (Aim2-/-) and n = 3 (WT) VSMC pools. d. mRNA levels of VSMC derived from AngII infused WT Bl6J ( n = 3 pools) and Aim2-/- (n = 3 pools) animals. ). A.u. arbitrary unit; relative expression of target gene was normalized against expression of housekeeping genes (Actb, Gapdh and B2m).
